# Realistic modeling of tumor cells

**DOI:** 10.18632/oncotarget.17122

**Published:** 2017-04-15

**Authors:** Kenneth L. van Golen

**Affiliations:** Center for Translational Cancer Research, Dept. of Biological Sciences, The University of Delaware and The Helen F. Graham Cancer Center, Newark, DE, USA

**Keywords:** Inflammatory breast cancer (IBC), tumor modeling, 3-dimensional cell culture, tumor emboli, lymphovascular invasion

Cell culture based assays have been a well-established and important method for the study of tumor cell behavior. The basis for tumor cell behavior, interactions and signaling have all been studied using *in vitro* cell culture techniques. This has also been the corner stone of drug discovery assays. Typically, cell culture-based assays have been performed as 2-dimensionsional (2D) monolayer culture. Increasing evidence suggests that 3-dimensional (3D) cell culture may be more accurate and realistic for modeling tumor cell behavior. This is particularly true for specialized models such as advanced tumor spheroid models [reviewed in 1]. Our own laboratory demonstrated that metastatic prostate cancer cells grown in a hyaluronic hydrogel-based culture system behaved in a manner more consistent with that observed *in vivo* [2]. Namely, the prevalence of hyaluronic acid in connective tissue, made for a more realistic environment and triggered the expression and localization of hyaluronan-mediated invasion [2].

Inflammatory Breast Cancer (IBC) is a phenotypically unique form of locally advanced breast cancer, which carries a poor prognosis [3]. IBC does not form a palpable mass in the breast and is hallmarked by lymphovascular invasion (LVI) into the dermal lymphatic vessels of the skin overlying the breast; the invading tumor cells form intralymphatic tumor emboli [4, 5]. The current standard therapy for treating IBC is aggressive and pluridisciplinary, including systemic chemotherapy followed by radical mastectomy, radiotherapy, hormone therapy for hormone receptor-positive tumors and anti-ERBB2 drug for ERBB2-positive tumors [6]. The tumor emboli are thought to be key to IBC tumor cell survival and metastasis.

Previous molecular and phenotypic studies of IBC utilized 2D culture, with a few 3D studies embedding the tumor cells in extracellular matrices such as Matrigel [7]. All together, these studies did not focus on the IBC tumor emboli. Our laboratory developed a novel and cost-effective system to accurately culture IBC emboli *in vitro*. In this system we mimic the physical and mechanical properties of the dermal lymphatic system [8]. Using hyaluronic acid, which is prevalent in the dermal lymphatic system, we mimic the dynamic viscosity (18-20.4 cSt) and pH (7.5-7.7) of lymphatic fluid. By applying oscillatory shear forces to the culture system, we mimic the dynamic fluid forces (0.64-12 dynes/cm^2^) of the lymphatic fluid under normal physical activity [8]. In this culture system, only tumor cells that are able to undergo LVI such as IBC (but not non-inflammatory breast cancer) or melanoma, readily form tumor emboli [8].

This emboli culture system has greatly aided the study of IBC by placing the tumor cells in an environment that stimulates the formation of emboli similar to what is observed in pathology samples taken from patients [8]. The current study by Arora et. al. identified high levels of anti-apoptotic proteins in IBC tumor emboli by adapting the culture system to high-throughput analysis. The simplicity of the model lends itself both to conventional molecular and phenotype analysis as well as a high-throughput approach. The growth of IBC cells as tumor emboli allow for a better representation of how the cells would behave *in situ*. Both published and unpublished molecular studies comparing IBC cells grown in 2D culture conditions with those grown in our 3D emboli system, demonstrate significant differences in gene expression and cellular behavior that is akin to what is occurring in the patient [8]. Several laboratories have utilized this 3D emboli culture system to study different biological aspects of IBC emboli biology with success.

Like other 3D culture systems, our system is constantly being refined to more accurately mimic a specific microenvironment. We anticipate that this system will be changed and enhanced over time leading to improved cell to cell interactions, interactions of the cell with the physical environment and formation of biologically-relevant cell structures. Together this will give us more relevant results for the study of IBC.
